# Platforms Exploited for SARS-CoV-2 Vaccine Development

**DOI:** 10.3390/vaccines9010011

**Published:** 2020-12-25

**Authors:** Shilu Mathew, Muhammed Faheem, Neeraja A. Hassain, Fatiha M. Benslimane, Asmaa A. Al Thani, Hassan Zaraket, Hadi M. Yassine

**Affiliations:** 1 Biomedical Research Center, Qatar University, Doha 2173, Qatar; shilu.mathew@qu.edu.qa (S.M.); fatiha@qu.edu.qa (F.M.B.); aaja@qu.edu.qa (A.A.A.T.); 2 Genetics & Genome Biology Program, The Hospital for Sick Children, Toronto, ON M5G 0A4, Canada; a08faheem@gmail.com; 3 Department of Biotechnology, Jamal Mohamed College, Tamil Nadu 620020, India; neeraja.anandamyle@gmail.com; 4 Department of Public Health, College of Health Sciences, Qatar University, Doha 2173, Qatar; 5 Department of Experimental Pathology, Immunology, and Microbiology, Faculty of Medicine, American University of Beirut, Beirut 11-0236, Lebanon; hz34@aub.edu.lb; 6 Center for Infectious Diseases Research, American University of Beirut, Beirut 11-0236, Lebanon

**Keywords:** SARS-CoV-2, Covid-19, vaccines, clinical trials, vaccine platforms

## Abstract

The novel severe acute respiratory syndrome coronavirus 2 (SARS-CoV-2) is the only zoonotic-origin coronavirus (CoV) that has reached the pandemic stage. The virus uses its spike (S) glycoprotein to attach to the host cells and initiate a cascade of events that leads to infection. It has sternly affected public health, economy, education, and social behavior around the world. Several scientific and medical communities have mounted concerted efforts to limit this pandemic and the subsequent wave of viral spread by developing preventative and potential vaccines. So far, no medicine or vaccine has been approved to prevent or treat coronavirus disease 2019 (COVID-19). This review describes the latest advances in the development of SARS-CoV-2 vaccines for humans, mainly focusing on the lead candidates in clinical trials. Moreover, we seek to provide both the advantages and the disadvantages of the leading platforms used in current vaccine development, based on past vaccine delivery efforts for non-SARS CoV-2 infections. We also highlight the population groups who should receive a vaccine against COVID-19 in a timely manner to eradicate the pandemic rapidly.

## 1. Introduction

Severe acute respiratory syndrome coronavirus 2 (SARS-CoV-2) is the causative agent of coronavirus disease 2019 (COVID-19) that emerged in Wuhan, China, in December 2019 [1,2]. Infection with SARS-CoV-2 is asymptomatic or results in mild flu-like symptoms in about 80% of the patients. However, people aged over 70 have long-term health conditions such as asthma or diabetes; people with a weakened immune system may suffer from severe illnesses like pneumonia and acute respiratory failure. COVID-19 could also be fatal for people with comorbidities [2,3,4]. To enter the host cell, coronavirus (CoV) utilizes its transmembrane spike (S) glycoprotein. First, the virus binds to a cell surface receptor, subsequently enters endosomes, and eventually fuses with the lysosomal membranes [5]. The S protein exists as a trimer with two domains, the S1 head and the S2 stalk on a mature virus. SARS-CoV-2 S1 subunit contains the receptor-binding domain (RBD), which primarily identifies angiotensin-converting enzyme-2 (ACE-2) as its receptor [6]. The RBD continuously shifts between a standing position to bind with the receptor and a lying-down position for immune evasion [7]. Furthermore, for membrane fusion, the SARS-CoV-2 spike requires to be proteolytically cleaved at the S1/S2 boundary in such a way that S1 dissociates, and S2 undergoes a structural change [8]. These features of SARS-CoV-2 entry contribute to its rapid spread, severe symptoms, and high fatality rates of infected patients [9,10]. As of 8 September 2020, SARS-CoV-2 had infected over 28 million people in 216 countries/territories, causing over 906 thousand fatalities [11]. The virus has a very high infection rate, making it more contagious than other CoV, including the severe acute respiratory syndrome coronavirus 1 (SARS-CoV-1) and the Middle East respiratory syndrome coronavirus (MERS-CoV) [12,13]. Concerned both by the alarming spread and the severity levels, the World Health Organization (WHO) declared COVID-19 a pandemic by 11 March 2020 [14]. Despite continuous efforts to contain the pandemic, there is still no vaccine nor an approved therapeutic cure for COVID-19. The current treatments rely on existing antiviral repurposed drugs combined with adjunct immune therapy, such as corticosteroids treatment [15]. Hence, the development of SARS-CoV-2 effective prophylactics and vaccines remains crucial [16,17,18]. With this background, many institutions and pharmaceutical companies have stepped forward to develop an effective and safe vaccine by the end of December 2021. This ambitious plan initially focused on 125 potential vaccine candidates but it has narrowed to 34 vaccine candidates (as of 8 September 2020) currently in clinical evaluation [19]. Given this, we describe the different approaches, proposed mechanisms, and status of the leading candidate vaccines developed against COVID-19 now in clinical trials.

## 2. Multiple Vaccine Platforms and Vaccines Currently in Clinical Evaluation

**Figure 1:**
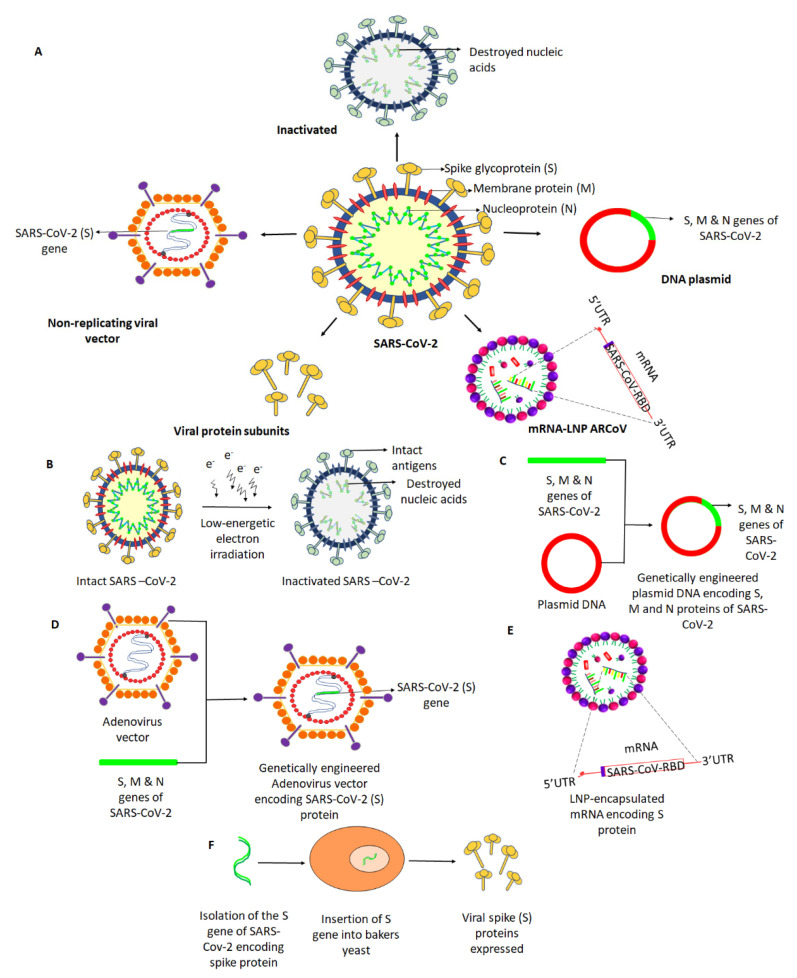
Different approaches for the development of vaccine candidates against SARS-Cov-2. (**A**) Potential vaccines under development involve five leading platforms (inactivated virus, protein subunit, DNA, RNA, and non-replicating viral vector), as depicted. (**B**) Intact SARS-CoV-2 is neutralized by treatment with radiation to cease its ability to infect and replicate, while preserving the induction of an immune response. (**C**) A plasmid DNA is genetically engineered with the *S*, *M*, and *N* genes of SARS-CoV-2 encoding the respective proteins that may facilitate an immune response. (**D**) A replication-defective Adenovirus (Ad) vector is genetically engineered to express SARS-Cov-2 spike (S) protein. (**E**) An mRNA (replication-defective) that encodes the S protein of SARS-CoV-2 is encapsulated in a lipid nanoparticle (LNP), which, when injected, induces the body cells to produce the spike protein and direct the immune response. (**F**) Spike protein-encoding (S) gene of SARS-CoV-2 was isolated and genetically engineered into a baker’s yeast, producing the spike protein antigens when grown. The produced S antigens can then be collected and purified.

To increase the chances of safe and effective vaccines for COVID-19, the WHO has facilitated the collaboration between many institutes and research communities across the world and has accelerated its efforts on a greater scale to evaluate different platforms for candidate vaccines [20]. The platforms currently exploited for SARS-CoV-2 vaccine are depicted in Figure 1A. As of September, the following vaccine platforms are in the final Phase II and Phase III of clinical trials [19]. Table 1 presents the current vaccine candidates in clinical evaluation, proposed by the WHO (8 September 2020).

### 2.1. Inactivated Viral Vaccine

An inactivated vaccine (whole killed virus) consists of virus particles whose replication has ceased but that retain the ability to induce an immune response [38]. Inactivated vaccines are synthesized by neutralizing a virus using heat radiation or chemicals such as β-propriolactone (Figure 1B). Inactivated vaccines can be produced at a large scale with reduced effort compared to other viral vaccines and would induce a robust immune response if used with proper adjuvants [38]. PiCoVacc (NCT04456595) is an inactivated virus vaccine developed by the Beijing-based Sinovac Biotech [39]. According to the investigators, this vaccine elicits neutralizing antibodies and could stimulate a ten-fold increase in antibodies against the virus’s spike protein in mice, rats, and macaques [27,40]. Concerning safety, it did not cause fever and weight loss; both appetite and mental state remained stable in the tested animals after immunization with PiCoVacc [27].

Moreover, histopathological evaluations of various organs, including lung, heart, spleen, liver, kidney, and brain from the tested animals demonstrated that PiCoVacc did not cause any notable pathology, especially in macaques [27]. Sinovac (Sinopharm) began Phase I and Phase II trials in April. The participants did not report any adverse effects in early clinical trials. The researchers found that the vaccine triggered neutralizing antibodies 14 days following vaccination with double dosage at day zero and 14. As of now, recruiting procedures have been started for a Phase III clinical trial with an estimated study completion date by October 2021 [39,41]. Besides, before the completion of the Phase III trial, Sinovac biotech has received emergency-use approval of this vaccine under a program in China to vaccinate high-risk groups and prevent possible new outbreaks. In this complete analysis of Phase I and Phase II clinical trials recently, Sinopharm also developed an inactivated form of the SARS-CoV-2 candidate vaccine (ChiCTR2000034780) and published its clinical trial data recently [40]. The researchers found that the vaccine elicited neutralizing antibodies while inducing mild pain at the injection site and fever in a small subset of vaccinated subjects [42]. Given these data, the company has already begun its clinical Phase III trials with expected completion by July 2021 [43].

### 2.2. Non-Replicating Viral Vector

Non-replicating viral vaccine platforms use primarily replication-defective viral vectors. These are based on a weakened common cold virus that readily infects human cells but is incapable of causing disease. Adenoviruses (Ad) are among the most heavily exploited non-replicating vectors that mimic a natural viral infection and induce the production of the target viral proteins inside host cells (Figure 1C) [44]. Adenovirus vector-based strategies are being developed to prevent or control an emerging infectious disease. Ad vector vaccines were developed against human immunodeficiency virus (HIV), ebola, and influenza and are under clinical evaluation [45]. To date, there is no Ad vector-based vaccine approved officially for human use. This platform’s limitations include pre-existing neutralizing antibodies against the vector, induction of inflammatory responses, sequestering of the vector in the liver and spleen, and immunodominance of the vector genes over transgenes [46]. As of recently, adenovirus type-5-vectored coronavirus (Ad5-nCov) (ChiCTR2000030906) against SARS-Cov-2 is the first vector-based vaccine in a Phase III clinical trial, developed by the Beijing Institute of Biotechnology and CanSino Biologics. This genetically engineered vaccine candidate acts as a vector for the expression of the SARS-CoV-2 spike protein that has been shown to induce a good immune response with protective efficacy [21]. Furthermore, the data of Phase I and Phase II analyses, indicated mild to moderate adverse events among the participants, and further investigation will be continued until 31 January 2021 [47]. In collaboration with Oxford University, the pharmaceutical company AstraZeneca is developing another non-replicating viral vaccine named ChAdOx1 nCoV-19 (NCT04324606.), also known as the Oxford vaccine [48]. The ChAdOx1 nCoV-19 vaccine consists of the replication-deficient simian adenovirus vector ChAdOx1, containing the full-length spike protein of SARS-CoV-2, with a tissue plasminogen activator leader sequence [6]. As per the preliminary findings, the candidate vaccine appears safe, tolerated, and induced high levels of neutralizing antibodies in the participants. Further, the study reported no serious adverse events related to ChAdOx1 nCoV-19 [6]. As of now, the study is in a Phase III clinical trial, with an expected release date by October 2021. Johnson & Johnson’s Janssen experimental COVID-19 vaccine (NCT04436276) is another vaccine based on a non-replicating adenoviral vector. The vaccine uses adenovirus serotype 26 (Ad26) to expresses the SARS-CoV-2 spike protein. The results from its experimentation in non-human primates (NHP) have recently been published [49]. Of note, the study demonstrated robust single-shot vaccine protection against SARS-CoV-2, with nearly a complete protective efficacy and optimal neutralizing antibody responses [49]. As of now, it is currently being evaluated in Phase I and IIa clinical trials, with an expected completion date by November 2023 [50]. Gam-COVID-Vac Lyo (NCT04437875, renamed Sputnik V) is another non-replicating-vector-based vaccine with a combination of two adenoviruses, Ad5 and Ad26, both engineered with the spike gene. It is developed by the Gamaleya Research Institute, and its Phase III trials were recently launched [51]. However, there are no reports on the effective dose, immune response, and efficacy from Phase I or Phase II trials against COVID-19 [52]. The study has started recruiting participants for a Phase III trial, with a very close completion date estimated in August 2020 [52].

### 2.3. RNA Vaccine

mRNA vaccines are new-generation vaccination strategies effective in activating the immune response in a way similar to that of natural infection. They are short synthetic viral mRNAs, used by the host to produce the target antigens. Further, the mRNA used in the vaccine is reportedly safe, as it cannot integrate into the host genome (Figure 1D). One such mRNA vaccine candidate is mRNA-1273 (NCT04283461), developed by Moderna, a Massachusetts-based biotechnology company [53]. This vaccine is synthesized using non-replicating RNA genetic material in a lipid nanoparticle (LNP) formulation, which encodes a prefusion-stabilized form of the spike protein of SARS-CoV-2 [31]. The vaccine directs the body cells to express the spike protein in its prefusion conformation to elicit an immune response. The study data indicate that this vaccine is well tolerated and prompted neutralizing antibody activity in healthy adults [31]. Regarding its safety, more than half of the participants reported fatigue, headache, chills, myalgia, or pain at the injection site, but no serious adverse effects were reported. Likewise, mild to moderate symptoms were reported among older adults in a recent report published by the NCT04283461 trial group [32]. Given these data, the company is forging on with a Phase IIa trial, with a target completion date by August 2021 [31]. BioNTech, in partnership with Pfizer and Fosun Pharma, proposed a vaccine candidate in two versions: BNT162b1, which encodes a secreted trimerized SARS-CoV-2 receptor-binding domain, and BNT162b2, which encodes a prefusion-stabilized membrane-anchored SARS-CoV-2 full-length spike [54]. Interim safety and immunogenicity data collected from Phase I clinical trials of BNT162b1 in younger adults had been reported earlier from the US and Germany, while BNT162b2 was associated with less systemic reactogenicity, especially in older adults [30]. At present, after Phase IIb and III trials of BNT162b2, an upcoming large clinical trial will focus on checking long-term immunity, with an expected release date by November 2022 [54]. Another mRNA vaccine candidate is ARCoV (Chictr2000034112), currently in its Phase III clinical trial, with expected release date in December 2022. The vaccine was proposed jointly by the Academy of Military Sciences, Suzhou Abogen Biosciences, and Walvax Biotechnology Company. The trial data indicated that the ARCoV vaccine induced high levels of neutralizing antibodies in mice and crab-eating macaques and induced protective T cell immune responses [55]. Moreover, the immune response to the spike protein’s receptor-binding domain will be explored [56].

### 2.4. DNA-Based Vaccine

DNA vaccination provides attractive approaches compared to viral vector-based, live vaccines due to DNA vaccine stability, simplicity, efficacy, and safety. DNA vaccines consist of plasmid DNA encoding antigenic viral proteins known to induce both B and T cell responses. In the field of DNA vaccination, substantial progress has been achieved concerning vaccine safety and efficacy [57]. The fundamental idea behind DNA vaccines is to induce immune responses against recombinant antigens encoded by genetically engineered DNA plasmids (Figure 1E). After immunization, the host cellular machinery facilitates the expression of plasmid-encoded genes, which leads to the generation of foreign antigens that can be processed and presented by both major histocompatibility complex (MHC) class I and II molecules. The immune system can recognize these host-synthesized foreign antigens, inducing a complete and adequate immunization [48]. INO-4800 is one such DNA vaccine currently in clinical trial.

Further, the INO-4800 vaccine targets the major surface antigen spike protein of the SARS-CoV-2 virus and is known to generate a robust binding and neutralizing antibody response in guinea pigs and mice [29]. The vaccine is introduced in the human body by a hand-held smart device called CELECTRA^®^, which uses a brief electrical pulse to reversibly open small pores in the cells to allow the plasmids to enter [58]. The study also detected these antibodies in the lungs of the vaccinated animals, which could be important in the protection against SARS-CoV-2. Another DNA vaccine, GX-19 (NTC04445389), administered via electroporation, is being developed by a Genexine-led consortium, the International Vaccine Institute, GenNbio, Korea Advanced Institute of Science and Technology, and Pohang University of Science and Technology. In view of a clinical study, initial experiments found a robust production of antibodies in mice and NHP models capable of neutralizing the novel CoV-2. Moreover, the adverse effects of vaccination and antigen-specific T cell immune response need to be assessed using various doses of GX-19; the proposed completion date is by June 2022 [59].

### 2.5. Protein Subunit

A protein subunit (adjuvanted recombinant vaccine) vaccine is based on antigens/viral components that best stimulate the human immune system without introducing viral particles (Figure 1F) [38]. It requires the insertion of adjuvants to elicit a protective immune response, because the antigen alone is incapable of inducing long-term immunity [60]. Based on the S protein, protein subunit vaccines are likely to activate antibodies that prevent virus binding and later fusion of membranes, thereby counteracting virus infection [6]. China’s Chongqing Zhifei Biological products developed a protein subunit recombinant protein vaccine (NCT04466085) that is into Phase II human trials. However, the investigators did not provide details of the outcome of the Phase I test of the experimental vaccine and have proposed a study completion date by December 2021 [61].

Within the context of global health, our knowledge about health challenges posed by pathogens potentially causing infectious disease epidemics and pandemics has increased. These challenges inspired efforts to curtail SARS-CoV-2 spread by developing vaccines. As of now, different platforms are widely used for vaccine development. These platforms have their advantages and disadvantages, with respect to vaccines ability to induce an effective immune response, manufacturing capacity, and users’ safety (Table 2). The viral vector-based vaccine technology employs either live (replicating but often attenuated) or non-replicating vectors. Viral vector-based vaccines could induce an immune response to the target antigen. Viral genomes can be manipulated to express an antigen of choice, enhancing their ability to accept relatively more insertions in the genome, and can be used to develop vaccines against various pathogens.

Additionally, viral vector vaccines could be delivered without added adjuvants and, depending on which vector is employed, antigen-specific cellular and humoral immune responses could be induced. High production has been achieved for commonly employed viral-based vaccines, supporting these technologies for pandemic settings [62]. However, so far, only one viral vector-based vaccine has been approved for use in humans. Dengvaxia is a recombinant Dengue vaccine based on yellow fever attenuated strain 17D, recently approved to prevent Dengue in previously infected individuals [62]. Further, several clinical trials have been done using viral vector-based vaccines, like that with vesicular stomatitis virus—Zaire ebolavirus (VSV-ZEBOV)—that has been shown to induce a protective response in humans [62]. Adenoviral vectors have many advantages. They are used to deliver genes in vivo, as most human cells express primary adenovirus receptors and secondary integrin receptors [63]. They are commonly used vectors in clinical trials globally and are being employed in more than 20% of all gene therapy trials. They allow the application of viral capsid modification strategies to increase their therapeutic characteristics and enhance virus targeting specificity [63]. AdVs are used to deliver engineered CRISPR/Cas9 systems to target cells and or tissues for genome engineering [64,65], which could not be achieved effectively through other viral systems.

Moreover, high-capacity adenoviral vectors (HC-AdVs) could deliver anti-PD1/PD-L1 immune checkpoint therapeutics for cancer treatment [69,70]. However, they also present challenges. The main obstacles to using viral vectors are immune system reactions, the vector packaging capacity, viral longevity, and viral contamination with helper virus (HV). Viral components removal and the emergence of high-capacity adenoviral vectors (HC-AdVs) considerably decreased the immune reaction, among other benefits. Immune reduction supports the prolongation of virus lifespan and prevents tissue damage and inflammation. A challenge related to HC-AdV prolonged expression is related to possible prior immunization against Ad. To overcome this concern, HC-AdV longevity could be optimized by encoding novel tetracycline-dependent (TetOn) regulatory elements [71]. With their expression regulated by doxycycline, HC-Ad-TetOn vectors showed better regulation and effectiveness than constitutively active HC-AdV, in the presence of an immune response [72]. The deficiency of specific viral genes enhanced the packaging capacity. However, the presence of the viral packaging machinery could pose challenges, as it limits the number of unique enzyme sites for transgene insertion. This concern was resolved using HD-AdVs with a unique transgene insertion site and preserving tissue specificity [73]. Moreover, viral-vectored vaccines require different manufacturing facilities and cellular systems, depending on the used virus. During synthesis, viruses might experience recombination, so precautionary measures need to be followed to keep cell cultures free of material leading to the emergence of recombined and uncharacterized pathogens [74]. More challenges are cellular barriers, viral targeting, and transport.

DNA-based vaccines have several advantages associated with their development and production. Nevertheless, they also have some disadvantages. One concern is the long-term persistence of DNA plasmids in host cells. Different preclinical studies demonstrated the existence of plasmid DNA for up to two years upon intramuscular injection, with lower but detectable expression and immunogenicity in a mouse model [75]. Additionally, the injection of bacterial DNA, sensed due to unmethylated CpG motifs, is linked with safety concerns, such as antibody generation against the injected DNA. Nonetheless, anti-DNA antibodies have not been detected in mice, rabbits, rats, or NHP [76]. Furthermore, cytokines or co-stimulatory molecules used to increase DNA immunogenicity could lead to unintended adverse effects by triggering cytokine expression and inducing generalized immune suppression, autoimmunity, or chronic inflammation. DNA vaccines have undergone clinical trials against a wide range of human pathogens like HIV, malaria, influenza virus, hepatitis B virus, herpes simplex virus, and respiratory syncytial virus.

As of now, no DNA-based vaccine is licensed for human use. However, different DNA-based vaccines have been licensed for veterinary applications, such as the equine vaccine against West Nile Virus. Due to their incredible versatility, these vaccines have been tested for their efficacy against recent pandemic threats, including Ebola, Zika (ZIKV), and MERS [76]. The first vaccine for the Ebola virus was DNA-based [77]. It promoted the expression of a viral glycoprotein (GP) to induce neutralizing antibodies as well as nucleoprotein as an antibody target and inducer of T cell responses. Based on preclinical results using the DNA vaccine, a Phase I (NCT00072605) clinical trial for the DNA-based Ebola vaccine was started in 2003 [78]. The study employed a trivalent DNA vaccine comprising plasmids encoding transmembrane deleted forms of a glycoprotein derived from two Ebola viruses and a nucleoprotein generated by Vical Inc [78]. The study confirmed safety, tolerability, and a specific antibody response to at least one of the three viral antigens in all subjects. However, the virus-neutralizing response was not detected in this trial. Another clinical trial in Phase I (NCT00605514) started in 2008–2009 and showed an elicited response to transmembrane deletions of a viral GP in the context of adenoviral delivery in NHPs [79,80].

Another clinical study in phase IB of two DNA vaccines that encode glycoproteins of Ebola virus and Marburg virus (MARV) demonstrated the safety of both vaccines [81]. An elicited immune response against a viral glycoprotein was observed in 80% of the subjects tested in the Phase IB study (NCT00997607) conducted in Uganda to establish the safety profile [81]. These vaccines were well tolerated, but the immune response to the Ebola virus and MARV was elicited in 50% and 30% of the subjects, respectively. Inovio developed a DNA vaccine for Ebola named INO-4212 (NCT02464670) and evaluated its efficacy and safety in a Phase I trial [82]. The study showed that 90% of the participants produced Ebola-specific antibodies [82].

Preclinical and clinical studies were also conducted to assess DNA vaccine protection ability against influenza viruses. Vaccines depend on the expression of plasmid-based hemagglutinin (HA), a viral surface glycoprotein and the main target of neutralizing antibodies against the influenza virus. In 2007, Phase I clinical trials of the DNA vaccine (NCT00709800 and NCT00694213) together with the lipid-based adjuvant Vaxfectin were conducted against influenza following the demonstration of its protective efficacy in mice and ferrets [83]. The vaccine was well tolerated and induced hemagglutination inhibition (HI) titers (a surrogate marker of neutralizing antibodies) of ≥40; protection was elicited in 67% and 20% of HA-only and trivalent groups, respectively [84].

With the emergence of the Zika virus (ZIKV) in 2016, once again, DNA-based vaccines were considered highly desirable and reached clinical trials. The first approach developed by Inovio employed precursor membrane and envelope (prM-E) proteins from Asian, African, and American strains modified to comprise an IgE signal peptide along with the removal of putative glycosylation sites [85]. The results showed an immunogenic and protective effect of this vaccine in a mouse model upon intramuscular vaccination followed by electroporation [62]. Likewise, virus antibodies, as well as T cell response, were observed in NHP. Two Phase I clinical studies were initiated based on these data, one in flavivirus-naive individuals (NCT02809443) and the second one in dengue virus-seropositive subjects (NCT02887482). Preliminary results from this study demonstrated that the vaccine induced neutralizing antibodies and was well tolerated in 62% of the participants [62].

The use of RNA-based vaccine technologies in humans is less well characterized in comparison to that of DNA-based or viral vector-based vaccines [62]. Vaccines based on self-amplifying mRNA encode different influenza antigens mixed with LNPs or oil-in-water cationic nanoemulsions (CNE) showed immunogenicity in ferrets. They facilitated viral replication in the upper respiratory tract because of influenza infection and protected against the virus in mice [86,87]. Likewise, a mouse model has demonstrated that the RNA replicon of modified dendrimer nanoparticles (MDNP) provides protection against influenza and Ebola infections. Moreover, it showed antibody production and CD8^+^ T cell response against Zika virus [88,89]. However, efficacy, stability, and tolerance of self-amplifying mRNA vaccines are not evident in humans. Numerous preclinical studies have shown that non-replicating mRNA vaccines could induce an immune response and safeguard against pathogens, such as EBOV, influenza, and ZIKV [62]. Studies have shown that a single low-dose of intradermal (ID) immunization with LNP (lipid nanoparticle)-encapsulated modified mRNA, encoding ZIKV prM-E glycoproteins, elicited neutralizing antibodies that showed protection in mice and NHPs [90,91]. Another study demonstrated that IM immunization with the same ZIKV vaccine showed enhanced titers of protective neutralizing antibodies and sterilizing immunity in mice [92,93]. LNP-encapsulated modified mRNA Ebola virus vaccine, encoding Ebola virus glycoprotein (EBOV GP), delivered IM (Intramuscular), demonstrated stimulated EBOV-specific IgG, neutralizing antibody response, and protection of guinea pigs from infection [94]. Further studies have shown that mRNA-based vaccines can elicit a protective immune response against influenza. Moreover, it was determined that ID intradermal-administered protamine complexed with a non-replicating sequence-optimized mRNA vaccine, encoding influenza HA, showed protection in mice against influenza H1N1, H3N2, and H5N1 and immunogenicity in ferrets and pigs [95]. Moreover, Phase-I clinical trials of H7N9 and H10N8 mRNA IM vaccines have shown no adverse effects. These mRNA vaccines have shown tolerance, protection, and elicited immunogenicity against H7N9 and H10N8 [96]. Table 3 reports a list of non-SARS-CoV-2 viral vaccine candidates in clinical development.

## 3. Previous Vaccine Development for CoV

SARS-CoV and MERS-CoV emerged in 2002–2003 and 2012–2013, respectively, causing acute respiratory tract infections in humans [100]. Although different platforms of vaccine development (live-attenuated, inactivated, vector-based, protein subunit-based, and nucleic acid-based) were evaluated for SARS-CoV and MERS-CoV, no vaccine has been approved. Several studies showed cellular immune response against a SARS-CoV live attenuated vaccine [101,102,103,104]. However, the use of live attenuated vaccines carries the risk of virus reversal to its wild type or virulent forms, especially in immune-compromised individuals [105,106,107]. Moreover, a few studies showed lung and liver inflammation, pro-inflammatory cytokine secretion, and neutrophil influx in animal models [108,109]. These vaccines are also not appropriate for an immunologically sensitive population, as they need multiple and high dosages, might allow virus reversal to its virulent form, or might induce a suboptimal response.

Compared to live attenuated vaccines, inactivated (non-replicating) vaccines are considered a safer option [110]. A formaldehyde-inactivated SARS-CoV vaccine adjuvanted with aluminum hydroxide preserved antigenicity, eliciting neutralizing antibodies in mice [111]. Another study showed that a UV-inactivated SARS-CoV vaccine elicited an immunogenic humoral response in mice. Further studies indicated humoral as well as mucosal immunity against an inactivated SARS-CoV vaccine in rhesus monkey [111]. However, the unpredictable immune response is one of the primary constraints of the inactivated vaccine [110]. Few studies have shown that inactivated vaccines are associated with adverse events, like inflammation, lung lesions, eosinophil infiltration, and weak or delayed immune response [111,112].

Several protein subunit-based vaccines were developed against the influenza virus, meningitis, hepatitis B, and pneumonia [113,114,115,116]. Studies have considered full-length or segmented coronavirus proteins for vaccines, such as the receptor-binding domain or full-length spike or envelop proteins [113,114,115,116,117,118]. A study showed that a fusion of protein containing spike protein residues 318–510 produced an efficient immune response in rabbits [119]. Another study showed that SARS-CoV S2 epitope peptide elicited antigenicity of the S2 protein [115]. Moreover, a MERS-CoV vaccine based on the receptor-binding domain demonstrated both humoral and cellular responses with the least dose of the antigen (1 µg) [120]. Protein subunit-based vaccines have the least chances of adverse effects. Since they lead to the production of a short antigen segment, they are safe compared to live attenuated and inactivated vaccines [113]. On the other hand, some reports showed inadequate or delayed immunogenic response against SARS-CoV because of lack of viral genetic material, but this could be overcome using a suitable adjuvant [114,116,118].

Nucleic acid-based vaccine are safe compared to live attenuated and inactivated vaccines [121,122]. One study showed that the SARS-CoV conserved nucleocapsid protein induced the production of CD8^+^ cytotoxic T lymphocytes and γ-interferon response [123]. Another study demonstrated that the injection of fragments of the SARS-CoV spike gene resulted in the production of cytotoxic T lymphocytes, IgG, and CD8^+^ T lymphocytes in rats between 3 and 7 weeks from the injection [124]. Likewise, a study has shown that T helper type-1 and type-2 immune responses against SARS-CoV were induced as a result of the combining DNA killed-virus vaccines [125]. Another DNA vaccine was designed to encode the S1 and S2 subunits and induced an immune response (a specific antibody) in mice [126]. The drawbacks were a limited immune response linked the specific or engineered genetic material, laborious genetic engineering, injection site pain, and required adjuvant for long-term immunity [124,127].

## 4. Target Groups to Receive Vaccines

Targeted immunization approaches are designed to increase the immunization level of populations prone to severe health conditions. Considering this, priorities related to the administration of COVID-19 vaccination are critical and must be decided in society’s best interest. The U.S. Centers for Disease Control and Prevention (CDC) has made a five-tier scheme of vaccination. The top tier includes critical healthcare and other workers [128]. Tiers two and three comprise people working in healthcare and having other essential jobs or those who are 65 years and older, live in long-term care facilities, or have medical conditions with an increased risk of developing COVID-19. Tier four and five include the general population. In addition, prisoners, meat packers, soldiers, and grocery store workers also need to be considered as their professions or environment dramatically increase their risk of being infected [128]. Studies have shown that pregnant women might be at increased risk for severe COVID-19 and should be given priority [129]. Therefore, timeliness of targeted immunization will ensure saving lives, protecting the healthcare system, and finally restoring the suffering global economy.

## 5. Conclusions

People around the world are facing one of the biggest challenges of their lives, trying to protect themselves against COVID-19. The pandemic has inadvertently affected the lives of people, health and education systems, and the economy. Vaccination remains the mainstay for mitigating the pandemic and restoring some normalcy in the world. Prior efforts in vaccine development (live attenuated, inactivated, vector-based, protein subunit-based, and nucleic acid-based), targeting other viruses, including MERS and SARS, have facilitated the development of a vaccine for COVID-19. Vaccination for COVID-19 may be essential to achieve herd immunity and limit virus spread. However, concerns arise regarding vaccine reactogenicity and long-term side effects. Although the progress is fast thanks to the scientific community’s robust efforts, the production of a safe, effective, and preventive vaccine to control the COVID-19 pandemic might take a few more months.

## Figures and Tables

**Table 1:** List of candidate vaccines for severe acute respiratory syndrome coronavirus 2 (SARS-CoV-2) under clinical evaluation.

Vaccine Platform	COVID-19 Vaccine Developer/Manufacturer	Type of Candidate Vaccine	Dosage	Number of Shots (with Time Interval)	Primary Study Adverse Side Effects	Number of Subjects	References
Non-replicating viral vector	University of Oxford/AstraZeneca (August 2020–October 2022)	ChAdOx1-S NCT04516746, NCT04540393	5 × 10^10^ vp (nominal ± 1.5 × 10^10^ vp)	**2** (0, 28 days); IM	Phase 3 Higher efficacy regimen used a halved first dose and standard second dose	Humans (40,000 participants)	[19,21,22,23,24,25]
	CanSino Biological Inc./Beijing Institute of Biotechnology (September 2020–July 2021/ September 2020–January 2022)	Adenovirus type 5 vector NCT04540419/ NCT04526990	5 × 10^10^ vp/0.5 mL	**1**; IM	Phase 3 Induced significant immune response in phase 2 trial	Humans (40,000/500 participants)	
	Gamaleya Research Institute (September 2020–May 2021)	Adeno-based (rAd26-S + rAd5-S) NCT04530396	Gam-COVID-Vac 0.5 mL	**2** (0, 21 days); IM	Phase 3 Induced antibody responses with no serious adverse events	Humans (40,000 participants)	
	Janssen Pharmaceutical companies (September 2020–March 2023)	Ad26COVS1 NCT04505722	5 × 10^10^ vp	**1**; IM	Phase 3 Occurrence of moderate symptoms after vaccination	Humans (60,000 participants)	
	ReiThera/LEUKOCARE/Univercells (August 2020–July 2021)	Replication defective Simian Adenovirus (GRAd) encoding S NCT04528641	5 × 10^10^ vp, 1 × 10^11^ vp or 2 × 10^11^ vp	**1**; IM	Phase 1 NA	Humans (90 participants)	
Inactivated	Sinovac (July 2020–October 2021)	Inactivated NCT04456595	3 µg/0.5 mL	**2** (0, 14 days); IM	Phase 3 NA	Humans (13,060 participants)	[19,26,27,28]
	Wuhan Institute of Biological Products/Sinopharm (April 2020–November 2021)	Inactivated ChiCTR2000031809	2.5 µg, 5 µg and 10 µg/dose	**2** (0, 14 or 0,21 days); IM	Phase1/2 Adverse reaction reported within 7 days of injection.	Humans (340 participants)	
	Beijing Institute of Biological Products/Sinopharm (July 2020–July 2021)	Inactivated ChiCTR2000032459	2 µg, 4 µg or 8 µg	**2** (0,14 or 0,21 days); IM	Phase1/2 Adverse reaction was reported within 7 days of the first inoculation in 49% of subjects. Grade 3 fever was reported but was self-limited.	Humans (1192 participants)	
	Institute of Medical Biology, Chinese Academy of Medical Sciences (November 2020–November 2021)	Inactivated NCT04412538, NCT04470609	50 U/0.5 mL, 100 U/0.5 mL and 150 U/0.5 mL	**2** (0,28 days); IM	Phase 1b/2b Occurrence of serious adverse events after vaccination	Humans (471 participants)	
	Research Institute for Biological safety problems, Rep of Kazakhstan (September 2020–December 2020)	Inactivated NCT04530357	0.5 mL of QazCovid-in^®^	**2** (0,21 days); IM	Phase 1/2 Occurrence of serious adverse events after vaccination	Humans (244 participants)	
	Bharat Biotech (November 2020–February 2022)	Whole-Virion inactivated NCT04473690	KBP-COVID-19,	**2** (0,14 days); IM	Phase 1/2 Occurrence of immediate adverse effects after vaccination	Humans (180 participants)	
RNA	Moderna/NIAID (July 2020–October 2022)	LNP-encapsulated mRNA NCT04470427	100 µg/dose	**2** (0, 28 days); IM;	Phase 3 M-RNA-1273 met its primary efficacy endpoint in the first interim analysis with efficacy of 94.5%	Humans (30,000 participants)	[19,29,30,31,32]
	BioNTech/Fosum Pharma/Pfizer (April 2020–December 2022)	LNP-mRNAs NCT04368728	10 µg/dose,	**2** (0, 28 days); IM	Phase 2/3 Local reactions	Humans (43,998 participants)	
	Curevac (September 2020–November 2021)	mRNA NCT04515147	4 µg/dose,	**2** (0, 28 days); IM	Phase 2 NA	Humans (691 participants)	
	Arcturus/Duke-NUS	mRNA NCT04480957	0.5 mL	**4**; IM	Phase 1/2 Severe adverse effects after 7 days of vaccination	Humans (92 participants)	
	Imperial College London (April 2020–June 2021)	LNP-nCoVsaRNA ISRCTN17072692	0.1 µg/dose,	**2**; IM	Phase 1 NA	Humans	
	People’s Liberation Army (PLA) Academy of Military Sciences/Walvax Biotech. (June 2020–December 2021)	mRNA ChiCTR2000034112	5 × 10^10^, 1 × 10^11^ and 1.5 × 10^11^ vp	**2** (0, 14 or 0, 28 days); IM	Phase 1 NA	Humans (168 participants)	
DNA	Inovio Pharmaceuticals/International Vaccine Institute (July 2020–February 2022)	DNA plasmid vaccine with electroporation NCT04447781	INO-4800 1 mg or 2 mg/dose using CELECTRA^®^2000,	**2** (0, 28 days); ID	Phase 1/2 NA	Humans (160 participants)	[19,29]
	Osaka University/AnGes/Takara Bio (June 2020–July 2021)	DNA plasmid vaccine + Adjuvant NCT04463472	1 mg or 2 mg/dose	**2** (0, 14 days); IM	Phase 1/2 Incidence of treatment-serious adverse effects	Humans (30 participants)	
	Cadila Healthcare Limited (July 2020–July 2021)	DNA plasmid vaccine CTRI/2020/07/02635	1 mg/dose	**3** (0, 28, 56 days); ID	Phase 1/2 NA	Humans (1048 participants)	
	Genexine Consortium (June 2020–June 2022)	DNA vaccine (GX-19) NCT0445389	GX-19,	**2** (0,28 days); IM	Phase 1/2 Incidence of solicited, and serious adverse events	Humans (210 participants)	
Protein subunit	Novavax (August 2020–November 2021)	Full-length recombinant SARS CoV-2 glycoprotein nanoparticle vaccine adjuvanted with Matrix M NCT04533399	SARS-CoV-2rS-5 µg + 50 µg Matrix-M1 adjuvant (co-formulated)	**2** (0, 21 days); IM;	Phase 2b NA	Humans (4400 participants)	[19,33,34,35]
	Anhui Zhifei Longcom Biopharmaceutical/Institute of Microbiology, Chinese Academy of Sciences (July-2020–December-2020)	Adjuvanted recombinant protein (RBD-Dimer) NCT04466085	25 µg/0.5 mL (per dose)	**2 or 3** doses (0, 28 or 0, 28, 56 days); IM	Phase 2 NA	Humans (900 participant)	
	Kentucky Bioprocessing, Inc (November 2020–February 2022)	RBD-based NCT04473690	Low and high doses of KBP-201 COVID-19	**2** (0, 21 days); IM	Phase 1/2 Solicited administration-site reactions after 7 days of vaccination Occurrence of adverse events	Humans (180 participants)	
	Sanofi Pasteur/GSK (September 2020–October 2021)	S protein (baculovirus production) NCT04537208	Formulation not defined	**2** (0, 21 days); IM	Phase 1/2 NA	Humans (440 participants)	
	Clover Biopharmaceuticals Inc./GSk/Dynavax June 2020–March 2021)	Native like Trimeric subunit Spike Protein vaccine NCT044405908	3 µg/dose	**2** (0, 21 days); IM	Phase 1 Incidence of solicited adverse events after 7 days of vaccination Incidence of serious adverse events	Humans (150 participants)	
	Vaxine Pty Ltd./Medytox (June 2020–July 2020)	Recombinant spike protein with Advax^TM^ adjuvant NCT04453852	S antigen 25 µg + 15 mg Advax-2 adjuvant/dose	**1**; IM	Phase 1 Incidence of adverse events after 7 days of vaccination	Humans (40 participants)	
	University of Queensland/CSL/Seqirus (July 2020–July 2021)	Molecular clamp stabilized Spike protein with MF59 adjuvant ACTRN1262000067493	5 mcg, 15 mcg, and 45 mcg/0.5 mL	**2** (0, 28 days); IM	Phase 1 NA	Humans (120 participants)	
	Medigen Vaccine Biologics Corporation/NIAID (October 2020–June 2021)	S-2P protein + CpG 1018 NCT04487210	MVC-COV1901	**2** (0, 28 days); IM	Phase 1 NA	Humans (45 participants)	
	Instituto Finlay de Vancunas, Cuba (August 2020–January 2021)	RBD + Adjuvant IFV/CR/06	Not specified	**2** (0, 28 days); IM	Phase 1 Serious adverse events measurement, daily after each dose	Humans	
	FBRI SRC VECTOR, Rospotrebnadzor July 2020–October 2020	RBD + Adjuvant NCT04527575	EpiVacCorona 0.5 mL/dose	**2** (0, 28 days); IM	Phase 1 NA	Humans (100 participants-Active, not recruiting)	
	West China Hospital, Sichuan University (October 2020–October2021)	Peptide ChiCTR2000037518	20 µg and 40 µg/0.5 mL	**2** (0, 21 days); IM	Phase 1 NA	Humans (120 participants-Active, not recruiting))	
Others (replicating viral vector)	Institute Pasteur/Themis/Univ. of Pittsburg CVR/Merck Sharp & Dohme (August 2020–October2021)	Measles-vector based NCT04497298	TMV-083	**1 or 2 doses** (0, 28 days); IM	Phase 1 NA	Humans (90 participants)	[19,36]
Others (VLP)	Medicago Inc. (July 2020–December 2021)	Plant-derived VLP adjuvanted with GSK or Dynavax adjs. NCT04450004	3.75 µg, 7.5 µg and 15 µg/dose	**2** (0, 21 days); IM	Phase 1 NA	Humans (180 participants, Active-not recruiting))	[19,37]

Covid-19, coronavirus disease 2019, LNP, lipid nanoparticle, INO, Inovio, GX, Genexine, KBP, Kentucky BioProcessing, TMV, Themis measles vaccine, VLP, virus like particle, GSK, GlaxoSmithKline, SU, RBD, RNA binding domain, MVC, Medigen vaccine biologics corporation, SU, Standardized Units, VP, viral particles, IM, Intramuscular, ID, Intradermal.

**Table 2:** Different vaccine platforms—pros and cons and examples of licensed non-SARS-CoV-2 vaccines for humans.

Vaccine Platform	Advantages	Disadvantages	Examples of Licensed Viral Vaccines Targeted for Humans	References
Viral vector-based	Exhibit highly specific gene delivery into the host cell with rigorous immune response. No infectious virus needs to be handled, shows significant preclinical and clinical data for many emerging viruses, including MERS-CoV.	Host’s immunity against the vector might negatively affect the effectiveness of the vaccine (depends on the vector chosen). The integration of the viral genome into the host genome may cause cancer.	JYNNEOS (smallpox/Monkeypox) ACAM2000 (smallpox) Adenovirus type 4 and type 7 vaccine, live, oral (febrile acute respiratory) Dengvaxia (Dengue)	[16,66,67,68]
Live attenuated	Develops long-lasting immunity High potency and pre-existing infrastructure used for several licensed human vaccines. Low-cost manufacturing.	Possible regression to virulence strain. Limited use in immunocompromised patients. Making infectious clones for attenuated coronavirus vaccine seed may be time consuming because of its large genome size. Extensive safety testing required.	ERVEBO (Ebola virus) MMR II (Measles, Mumps and Rubella)	[16,66,67,68]
Inactivated	Stable and safe compared to live attenuated virus platform. Pre-existing technology and infrastructure required for development are available. Can be used in immunocompromised patients. Has already been tested in humans for various diseases such as SARS-CoV-1 and adjuvants can be used to increase immunogenicity.	Requires booster doses to maintain immunity. Large amount of virus needs to be handled and antigen integrity needs to be confirmed. Low production titer.	Poliovax (Polio) Flucelvax Quadrivalent (Influenza) Ixiaro (Japanese Encephalitis) Imovax (Rabies)	[16,66,67,68]
RNA	Handling of infectious viral particle is not required. Low-cost and ease of manufacturing. Translation of mRNA occurs in the cytosol of the host cell thus reducing the risk of integration into the host genome.	May have low immunogenicity due to instability. Safety issue with reactogenicity have been reported for various RNA based vaccines. Multiple doses may be required.	None	[16,66,67,68]
DNA	Handling of infectious viral particle is not required. Ease of manufacturing. The synthetic DNA is temperature stable and cold-chain free	The titer remains low, even though it elicits both cytotoxic and humoral immunity. Potential integration to human genome causes abnormalities.	None	[16,66,67,68]
Protein subunit	Can be used in immunocompromised patients. Does not involve any live component of the viral particle	Low immunogenicity. Conjugation leads to batch-wise variation.	PedvaxHIB (*Haemophilus influenzae* type b) Engerix-B (Hepatitis B) Recombivax HB (hepatitis B)	[16,66,67,68]

MERS-CoV, Middle East respiratory syndrome coronavirus, SARS-CoV, severe acute respiratory syndrome-corona virus.

**Table 3:** Non-SARS-CoV-2 viral vaccine candidates in clinical development.

Study Start	Vaccine and Delivery	Study Outcomes	Reference
NCT00072605 October 2003	Ebola-DNA trivalent; NF inj.dev.IM 2–8 mg in week 0, 4, and 8 Antigens: GPΔTM EBOV GPΔTM SUDV NP	Phase I Acceptable safety profile Specific antibody response to at least 1/3 antigens in all subjects. Specific CD8^+^ T cell response in 30% subjects. No detectable virus neutralizing responses.	[62,79,97]
NCT00605514 January 2008	Ebola-DNA Mono or bivalent; NF inj.dev. IM 4 mg in week 0, 4, 8 Antigen: GP MARV GP EBOV + GP SUDV	Phase I Acceptable safety profile. Specific antibody responses against one of the GP’s at week 12 in 80% of subjects. CD8^+^ T cell response in some of the subjects.	
NCT02464670 May 2015	Ebola-DNA mono-, bi-, or trivalent; IM or ID + EP in 2 or 3 doses 0.8–4 mg GP; 0.2–1 mg IL 12 Antigen: GP EBOV pre 2013 and/or GP EBOV 2014 and IL 12 in trivalent vaccine	Phase I Acceptable safety profile Specific antibody responses in 88% (IM) and 95% (ID) of participants.	
NCT00709800 and NCT00694213 August 2007	Influenza H5N1-DNA mono- or trivalent; needle or NF inj.dev. IM 0.1–1 mg in week 0,3 Antigen: HA of/Vietnam/1203/04 HA + NP + M2	Phase I Acceptable safety profile Hi titers ≥40, in 47–67% (HA only) and 0–20% (HA + NP + M2) of participants Responses against HA unaffected by injection method.	[62,98]
NCT00973895 August 2009	Influenza H1N1-DNA Monovalent; NF inj.dev. IM 4 mg in week 0,4,8 Antigen: HA of A/California/04/2009	Phase I Acceptable safety profile HI titers ≥40 in 30% of DNA vaccinated subjects DNA + licensed vaccine HI titers ≥40 in 72% T cell response in 25% of subjects	[62,99]
NCT02809443 July 2016	Zika–DNAmonovalent; ID + EP 1 or 2 mg in week 0,4,12 Antigen: Consensus prM-E; IgE SP; removed glycosylation site	Phase I VNTs in 62% of the participants (Vero cell assay) Protection of 92% of mice by passive serum transfer in challenge mode (IFN α/β receptor knockout)	[62,87,88,90]
NCT02840487 August 2016	Zika–DNA monovalent; needle of NF inj.dev. IM 4 mg in 2 or 3 doses Antigen: prM-E; JEV SP (VRC5283) prM-E; JEV SP and S/TM (VRC5288)	Phase I/Ib Humoral and T cell responses induced VNTs in 60–100% of subjects 4 week after the final vaccination Antibody responses 100% in VRC5283 participants in NF inj, in split doses group; best T cell and VNT responses	
NCT03014089 December 2016	Zika–RNA mRNA 1325, modified nucleotides; LNP formulated, Antigen: prM-E polyprotein	Phase I/II	
NCT03076385 December 2016	Influenza H10N8–RNA mRNA 1851, modified nucleotides; LNP formulated, Antigen: HA of H10N8 A/Jiangxi-Donghu/346/2013	Phase I HI titers ≥40 in 100% subjects at day 43 MN ≥20 in 87% at day 43	[62,96]
NCT03345043 May 2016	Influenza H7N9–RNA mRNA 1440, modified nucleotides; LNP formulated, Antigen: HA of H7N9 A/Anhui/1/2013	Phase I Results pending	[62,96]
NCT02344407 October 2014-November 2015	Ebola-viral vector (non-replicating) Single dose IM 2.5 × 10^10^, 5 × 10^10^, 1 × 10^11^ VP Antigen: GP EBOV (1976)	Phase I/II Serious adverse events within 12 months after inj. in 8.0% (40/500) of participants (9.4% in rVSV-ZEBOV. Antibody responses in 70.8 and 63.5% of the participants at 1 and 12 months, respectively (83.7 and 79.5 % for VSV-ZEBOV)	[62,77]

Ad5, Adenovirus 5; EBOV, Ebola virus; ICS, Intracellular staining; GP, Glycoprotein; PFU, Plaque-Forming Unit; EP, Electroporation; SUDV, Sudan virus; VSV, Vesicular stomatitis virus; VNT, Virus neutralization titers; VP, Virus particles; GPΔTM, Glycoprotein-delta transmembrane domain; HI, Hemagglutination inhibitors; HA, Hemagglutinin; IL 12, interleukin 12; MARV, Marburg virus; JEV, Japanese encephalitis virus; ID, intradermal; NP, nucleoprotein; VNT, virus neutralization titers; prM-E, pre membrane envelope; SP, signal peptide; S/TM, stem and transmembrane regions, NF.inj.dev, Needle-free injection device.
